# How do emotion regulation strategies influence the way personality affects obsessive-compulsive symptoms?

**DOI:** 10.1192/j.eurpsy.2023.528

**Published:** 2023-07-19

**Authors:** M. B. Couto, I. Araújo, M. Picó-Pérez, P. Morgado

**Affiliations:** ^1^Clinical Academic Center - Braga, Braga, Portugal; ^2^Life and Health Sciences Research Institute (ICVS), University of Minho, Braga, Portugal; ^3^ICVS/3B’s, PT Government Associate Laboratory, Braga/Guimarães, Portugal, Braga; ^4^Hospital da Senhora da Oliveira Guimarães, Guimarães; ^5^Life and Health Sciences Research Institute (ICVS), University of Minho, Braga, Portugal

## Abstract

**Introduction:**

Obsessive-Compulsive Disorder (OCD) is a chronic disabling condition, with considerable lifetime prevalence. There are interindividual differences regarding personality dimensions and how they affect obsessive- compulsive (OC) symptomatology. Furthermore, there is a connection between OC symptoms and the use of maladaptive emotion regulation strategies (expressive suppression) instead of using more cognitive reappraisal.

**Objectives:**

Explore the relationship between personality, emotion regulation strategies and OC symptoms by testing a path analytic model in a sample of healthy participants and in a sample of OCD patients.

**Methods:**

Two samples of participants were utilized. Sample 1 consists of 787 healthy participants from the general Portuguese population. Sample 2 is composed of 33 OCD patients and 32 Healthy Controls (HC). Participants completed different scales: Emotion Regulation Questionnaire (ERQ), Neuroticism-Extraversion-Openness Five-Factor Inventory (NEO-FFI) and Obsessive-Compulsive Inventory-Revised (OCI-R), through online surveys (sample 1) or a clinical interview (sample 2). These questionnaires were then analyzed with a path-analytic approach.

**Results:**

In sample 2, we found significant differences between OCD patients and HC in every OCI-R subscale, except Hoarding and Neutralizing. In the NEO-FFI, OCD patients scored higher on Neuroticism and lower on Extraversion. No significant differences were found regarding the ERQ. Relatively to sample 1: path analysis results showed that 13,4% of the variance of OC symptoms was explained by the best-fitting model. Only Neuroticism and Extraversion were directly associated with higher OCI-R Total scores, whereas Agreeableness predicted less OC symptoms. The use of Expressive Suppression was associated with more OC symptomology, but no significant connection was found with Cognitive Reappraisal. Regarding sample 2, no model was found, showing no modifying effect of emotion regulation strategies on OC Symptoms.

**Conclusions:**

There is a deep-rooted interconnection between personality and emotion regulation regarding OC symptomatology in a sample of healthy participants but no effect of emotion regulation was seen regarding OCD patients.

To sum up, promising results were obtained and it could be an important field for the OCD in terms of diagnostic, severity and treatment.

**Disclosure of Interest:**

None Declared

